# Decreased salivary α-amylase activity responding to citric acid stimulation in Myasthenia gravis with malnutrition

**DOI:** 10.1371/journal.pone.0269621

**Published:** 2022-06-15

**Authors:** Ye Huang, Wen-kai Wang, Xiao-mei Zheng, Long Yang, Li-hui Wang, Xiang-hong Qiu, Long-hui Chen, Ru-liu Li, Chuan-quan Lin

**Affiliations:** 1 Science and Technology Innovation Center, Guangzhou University of Chinese Medicine, Guangzhou, PR China; 2 Pi-Wei Institute, Guangzhou University of Chinese Medicine, Guangzhou, PR China; 3 First Affiliated Hospital of Guangzhou University of Chinese Medicine, Guangzhou, PR China; University of Pisa, ITALY

## Abstract

**Objectives:**

Malnutrition, defined according to Nutritional risk screening (NRS 2002), is commonly observed in patients of Myasthenia gravis (MG), a neuromuscular disorder manifested by varied degrees of skeletal muscle weakness. Because biochemical composition of saliva changes in correspondence to alterations in nutritional status, we tested our hypothesis that a certain saliva component(s) might serve as a biomarker(s) for nutrition status of MG, particularly for those MG patients with high risk of malnutrition.

**Materials and methods:**

60 MG patients and 60 subjects belonging to the healthy control group (HCG) were enrolled in this case-control study. The salivary α-amylase (sAA) activity, salivary flow rate (SFR), pH, total protein density (TPD), and the concentrations of chloride and calcium ions in MG group with or without malnutrition were measured before and after citric acid stimulation. Thereafter, the relationship between sAA activity and BMI was determined in MG and HCG.

**Results:**

Compared with HCG, more patients with malnutrition, increased TPD and chloride and calcium concentrations but decreased pH value and SFR both before and after acid stimulation, as well as reduced sAA activity, pH and TPD responses to acid stimulation. MG with malnutrition showed decreased sAA activity and TPD responding to acid stimulation compared with those without malnutrition. Compared with normal BMI, sAA activity response to acid stimulation was reduced in low BMI. There was a significant strong positive correlation between the ratio of sAA activity and BMI in MG.

**Conclusions:**

Salivary biochemical characteristics are abnormally altered in MG with malnutrition. Altered sAA activity responding to acid stimulation was associated with malnutrition.

**Clinical relevance:**

Decreased sAA activity responding to acid stimulation can reflect malnutrition state and may be one potential screening marker for MG patients with high risk of malnutrition.

## Introduction

Myasthenia gravis (MG), caused by a breakdown in the normal communication between nerves and muscles, is characterized by weakness and rapid fatigue of muscles under voluntary control. It has been reported that up to two-thirds of individuals with MG suffered from dysphagia was attributable to the fatigue of muscles involved in chewing and swallowing [1, 2], which in turn lead to deleterious complications, such as malnutrition [3], inappetence, and reduced quality of life [4]. Inappetence and malnutrition are commonly associated with long-term usage of glucocorticoids and immunosuppressants in MG. Several studies have reported that malnutrition of MG patients was closely related to long hospital stay, high morbidity and mortality, and increased economic burden [5, 6]. The nutrition status is associated with the incidence and prognosis of MG patients with complications of respiratory failure [7]. Malnutrition of MG patients has also been associated with increased risk of dyspnea [8]. Preoperative nutrition support to MG patients has been shown to be effective in reducing postoperative complications, as well as lowering the risk of postoperative infectious complications and shortening hospital stays [9]. Therefore, it is imperative to perform routine screening of nutrition status in MG patients to identify those patients with malnutrition. To date, a standard diagnostic method(s) for routine nutrition status measurement is unavailable.

Currently, two types of tools have been proposed for screening nutritional risk, namely simple tool and the multidimensional tool, respectively [10]. The simple tool screens for malnutrition by taking into considerations of laboratory tests and anthropometric measures, including body mass index (BMI), serum albumin, and percentage of recent weight loss. Estimation of food intake and disease severity are necessarily included in the factors of the nutritional evaluation, because deterioration of nutritional status is most likely linked to several contributing factors concerning food supply, apart from the disease process itself [11]. Hence, the multidimensional tool offers a more comprehensive assessment of nutrition status by assessing a variety of factors, including BMI, body weight loss and dietary intake, as well as disease severity and comorbidity. One commonly used multidimensional tool is known as the Nutritional Risk Screening (NRS2002) that is widely used in hospitals [12]. Although these tools are relatively rapid and easy to use, they are not always applicable to bedridden MG patients who are often too weak to stand up for body-weight measurements. Moreover, because determination of serum albumin is a relatively invasive procedure that does not allow multiple and frequent sampling, it is desirable to develop a non-invasive and cost-effective method for timely identifying patients who require nutritional support.

Saliva, a liquid secreted from salivary glands, contains compounds that also present in blood. and, to certain extent, reflects nutritional state and metabolic variations of the body [13]. Additionally, senses of taste and smell, dental status, and saliva flow are considered as important contributing factors for the maintenance of a good nutritional status [14, 15]. Numerous studies have shown that patients with reduced salivary secretion suffered from taste and smell disorders [16], and nutritional status was impaired in patients with taste and smell abnormalities [17]. Moreover, salivary compositions are closely associated with nutritional status, where some elements in saliva, such as protein, minerals and vitamins, were changed with alteration in nutritional status [18, 19]. Our prior data have shown that salivary α-amylase (sAA) activity, in response to citric acid stimulation, reduced significantly in low-BMI children and chronic non-atrophic gastritis patients [20, 21]. As mentioned, presently available full nutritional assessments are not only cumbersome but also invasive. Thus, it has been suggested that, saliva could be a reliable and useful non-invasive surrogate for assessing nutritional status [22].

To date, the specific salivary biochemical characteristics and their relationships with malnutrition in MG patients remain largely unknown. Herein, we hypothesize that malnutrition in MG potentially leads to altered salivary secretion, saliva composition and metabolism. To assess the malnutrition state and develop potential marker of screening for nutritional risk in MG patients, we studied the salivary biochemical characteristics in MG patients, especially patients with malnutrition and varying BMI, with the purpose to assess the salivary biochemical changes in MG patients under both pre-citric acid and post-citric acid stimulation, and the association between the compositions of saliva and malnutrition in MG patients. The present data clearly indicate an abnormal alternation of salivary biochemical characteristics in MG patients and demonstrated that reduced sAA activity, in response to citric acid stimulation, can reveal malnutrition status. These findings suggest strongly that the acute sAA activity, in response to citric acid stimulation, can be used a convenient tool in screening of nutritional risks in MG patients, which is important in timely and effectively achieving prevention of detrimental weight loss, improving health outcomes, and reducing mortality.

## Material and methods

### Participant enrollment

60 patients with MG in stage II were enrolled at The First Affiliated Hospital of Guangzhou University of Chinese Medicine from October 2015 to July 2016, and 60 healthy subjects were recruited as healthy control group (HCG) at Guangzhou University of Chinese Medicine. The diagnosis standard was confirmed by presentation of an EMG amplitude decrement on repetitive stimulation and/or by the presence of anti-AChR antibodies in the blood [23]. MG was classified according to Osserman’s classification set by the committee of the American myasthenia gravis foundation [24]. The duration of illness was ranged from 6 months to 24 months for the MG group, in order to minimize variation due to a broad disease course range. The healthy participants were defined as normal subjects without any serious symptoms or diseases, such as pain, drooling, inappetence, vomit, diarrhea, digestive system diseases, cardiovascular diseases, endocrine system diseases, and immunological diseases. And the potential healthy participants were recruited via advertisements in newspapers, through flyers posted in the campus of Guangzhou University of Chinese Medicine. Finally, 60 healthy participants were recruited from 92 initial participants.

The participant inclusion criteria were shown as followed: (i) age range of 18–50 years, (ii) willingness to participate in the study, and (iii) fit well with the diagnostic criteria for MG and healthy subjects. Participants who presented with any of the following categories were excluded: (i) pregnant and nursing women; (ii) oral disease, including periodontal disease and caries; (iii) autoimmune, infectious, or malignant disease; (iv) recent operation or trauma; (v) history of alcohol consumption and smoking; and (vi) drug use 7 days prior to the study, which directly affect salivary secretion, such as adrenergic agonists, cholinesterase inhibitors and antidepressants drugs.

### Ethical approval

This study was conducted according to the guidelines laid down in the Declaration of Helsinki and all procedures involving human subjects/patients were approved by the Academic Ethics Committee of Guangzhou University of Chinese Medicine (No. 2015-10-20). All of the participants provided written informed consent to participate in the study. Furthermore, our present report conformed to the Strengthening the Reporting of Observational Studies in Epidemiology (STROBE) guidelines.

### Sample collection and pretreatment

According to our prior optimized saliva collection method [25], we collected whole saliva from each patient at a constant temperature, in a bright and quiet environment at 7:00 AM–8:00 AM to minimize diurnal variation. All subjects were required to refrain from eating or drinking other than water 1 h before sample collection, and were comfortably seated under resting conditions for over 30 min. The whole saliva which sampled at rest state was collected by gently rotating swab of Salivette (Sarstedt, Nümbrecht, Germany) at a certain speed (six times per minute) for 1.5 min. Thereafter, the sample was placed in centrifuge tube and centrifuged (4°C, 4440 × g, 10 min) to obtain the supernatant liquid as non-stimulation salivary sample. Gustatory stimulation was achieved by placing a square filter paper (1 × 1 cm; Hangzhou Special Paper Co., Ltd., Hangzhou, Zejiang, China model 102) with 10% citric acid on the upper tip of the tongue for 30 s. Before collection, subjects were required to raise their tongue tips, which were then swabbed and dried with a cotton swab to prevent the retained citric acid from affecting the salivary pH value. Afterward, the stimulated saliva samples were collected with Salivette by the same procedure as that of non-stimulation saliva samples described previously. All the saliva samples were frozen at -80°C for subsequent measurement. And there was no complaint among all participants during sampling.

### Salivary indices test

Salivary flow rate (SFR, mL/min) was immediately detected after sample collection by gravimetric method [26]. Generally, the weights of tube or salivette before and after the whole saliva collection were defined as m1 (g) and m2 (g), respectively, and the collection time was recorded as t (min). The SFR value was obtained through the formula, where SFR (mL/min) = (m2-m1)/ (1 × t), where the whole salivary density was defined as 1.0 g/mL in accordance with a prior study [27]. Salivary pH value was measured immediately after saliva collection with the laboratory pH meter (FE20, METTLER TOLEDO Ltd., Greifensee, Switzerland). The sAA activity was detected through the enzymatic hydrolysis of a chromogenic substrate (maltose; Sigma Aldrich (Shanghai) Trading Co. Ltd. (China), Shanghai, China Product Number M5885) [28]. The substrate is cleaved by sAA and α-glucosidase to release ρ-nitrophenol, which has an absorption peak at 405 nm (yellow). The sAA activity was determined by measuring absorbance at 405nm by an ultraviolet spectrophotometer (UV mini-1240, Shimadzu, Kyoto, Japan) per unit time (ΔA/min) and was expressed as U/mL. The intra-assay and inter-assay relative standard deviations were 3.92 and 4.45, respectively. One unit (U) was defined as the amount of enzyme that is required to liberate 1.0 mg of maltose from starch in 3 min at pH 6.9 and37°C. The concentrations of salivary total proteins, salivary calcium (Ca^2+^), and chloride (Cl^-^) were measured according to the manufacturers’ instructions of the BCA Protein Assay Kit(CWBIO, Lot: 00101504, Beijing Com Win Biotech Co., Ltd., Changping, Beijing, China), QuantiChrom™ Calcium Assay Kit, and QuantiChrom™ Chloride Assay Kit (Bio Assay Systems, QuantiChrom™ Calcium Assay Kit (Lot: DICA-500) and QuantiChrom™ Chloride Assay Kit (Lot: DICL-250), Bio Assay Systems, Hayward, CA, USA), respectively. All analysis was carried out in a blinded manner.

### Screening for nutritional risk

Before sample collection, all participants were required to complete screening for nutritional status with the Nutritional Risk Screening (NRS 2002) method [29]. All participants are scored in each of the two components (i) malnutrition and (ii) disease severity, depending upon whether they are absent, mild, moderate or severe, giving a total score 0–6. Nutritional status of all participants was divided into two groups according to their NRS2002 scores; (i) the M-group with a total score of ≥ 3 was considered being nutritionally at-risk, and (ii) the N-Group with a total score of < 3 was considered not-at-risk.

### Statistical analysis

After the Shapiro test for normality and Homogeneity of variance test, the data between MG and HCG, as well as between MG patients with and without malnutrition were used a 2×2 mixed ANOVA to compare the differences of each test index before and after acid stimulation in the same group, before and after acid stimulation in different groups. The Mann-Whitney U test for between-group comparisons or the Wilcoxon signed-rank test for within-group comparisons were performed otherwise. Categorical differences between the two groups were tested using the Chi-square test or Fisher’s exact test when expected frequencies are less than 5. The correlation analysis was conducted between indexes by Spearman Correlation Analysis. The data were expressed as mean ± standard deviation of the mean. Analysis was performed with Statistical Product and Service Solutions (SPSS), Chicago, Illinois, USA 18.0, and the significant level was set at *P*< 0.05. Two-sided significant tests were used throughout the analysis.

## Results

### Clinical information in participants

There was no statistically significant baseline difference between MG and HCG in gender, age, and heart rate (Table 1). Meanwhile, the average body mass index (BMI) of MG was significantly lower than that of HCG (*p* < 0.01). The sample was divided into two groups according to their MNA scores. The M-group included 43 participants classified as nutritionally at-risk (NRS score ≥ 3), and the N-Group was made up of 17 participants not considered to be nutritionally at-risk (NRS score < 3) in MG. Higher rate of malnutrition was observed in MG group than HCG group (71.46%, 43/60 vs 0%, 0/60; *p* < 0.01).

**Table 1:** Clinical information for all participants.

	N	Gender		Age (years)	Heart rate	BMI (kg/m^2^)	Nutritional status (%)	
		Male	Female				M-group(NRS≥3)	N-group(NRS<3)
HCG	60	27	33	30.62±5.76	75.4±0.8	21.19±1.58	0(0/60)	100.0(60/60)
MG	60	24	36	42.48±9.25	74.8±0.9	18.57±2.04*	71.67(43/60) *	28.33 (17/60)

Note

*p<0.01, compared with HCG by unpaired t-test when variances are not significantly different or by Mann-Whitney U test when variances are significantly different. Data are expressed as mean±SD. The Fisher’s exact test was used to compare frequency numbers of the M-group in MG with observed data in HCG.

### Salivary biochemical characteristics pre-and post-acid stimulation

For MG, when compared with HCG, the concentrations for total protein density (TPD), calcium (Ca^2+^) and chloride (Cl^−^) were significantly increased, whereas pH value and SFR were decreased (*P*<0.01) under both pre- and post-acid stimulation conditions (Table 2).

**Table 2:** Salivary biochemical characteristics under pre- and post-acid stimulation conditions.

		N	sAA activity (U/mL)	pH value	SFR(mL/min)	TPD(mg/mL)	[Cl^−^] (mg/dL)	[Ca^2+^] (mg/dL)
HCG	Pre-	60	505.39±308.33	6.75±0.33	1.30±0.48	1.23±0.79	51.46±26.45	4.24±3.05
	Post-	60	542.04±284.39	6.93±0.27*	1.65±0.58*	0.90±0.62*	48.25±23.74	4.46±2.29*
MG	Pre-	60	575.14±273.03	6.38±0.49†	0.96±0.51†	2.02±1.22†	88.01±44.95†	10.73±7.58†
	Post-	60	450.68±249.96*	6.37±0.78§	1.18±0.59*§	1.21±0.68*§	72.01±33.78*§	11.36±7.26§

Note

*p<0.01, compared with the value at pre-acid stimulation in the same group by 2×2 mixed ANOVA when variances are not significantly different or by Wilcoxon signed-rank test when variances are significantly different

^†^*p*<0.01, compared with the value at pre-acid stimulation compared with the value at pre-acid stimulation in HCG by 2×2 mixed ANOVA when variances are not significantly different or by Mann-Whitney U test when variances are significantly different

^§^*p*<0.01, compared with the value at post-acid stimulation in HCG by 2×2 mixed ANOVA. Data are expressed as mean±SD.

Compared with pre-stimulation condition in the same group, the levels of SFR, pH value and Ca^2+^concentration were increased in HCG (*p*<0.01), while sAA activity and Cl^−^ concentration were decreased significantly (*p*<0.01) in MG after citric acid stimulation. Compared with pre-stimulation condition in the same group, SFR was increased, but TPD was decreased (*p*<0.01) in both HCG and MG. To sum up, the abnormal changes in salivary biochemical characteristics were observed in MG under both pre- and post-acid stimulation conditions.

### Percent changes of salivary indices after acid stimulation

**Fig 1:**
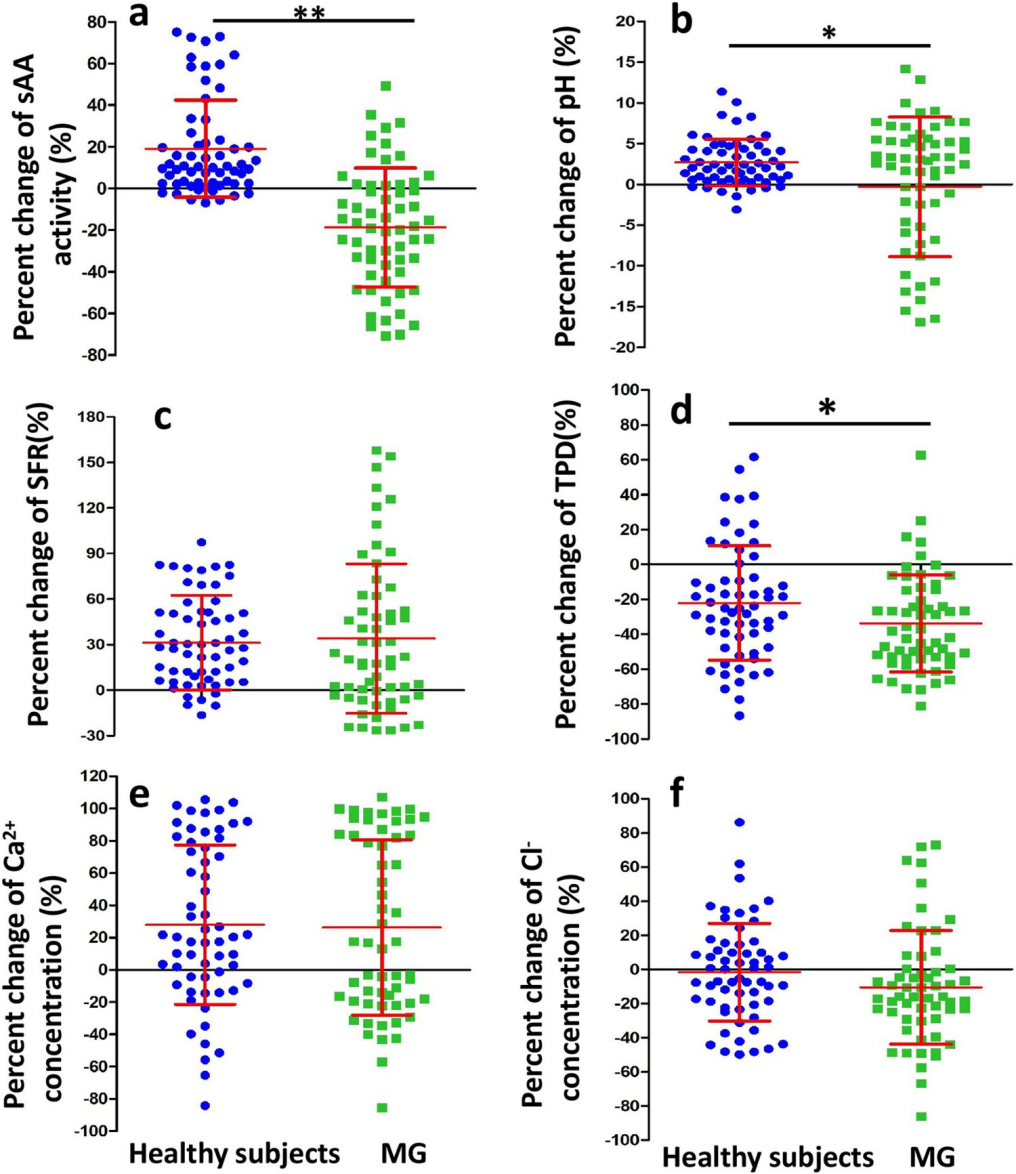
Salivary indicator changes after acid stimulation in HCG and MG. **(a)**: sAA activity, **(b)**: pH value, **(c)**: SFR, **(d)**: TPD, **(e)**: Ca^2+^ concentration, **(f)**: Cl^-^ concentration. Percent change reflects the extent of salivary secretion respond to acid stimulation and is calculated as [(value post-acid stimulation)—(value pre-acid stimulation)]/(value pre-acid stimulation) × 100%. The independent sample t test was applied at**p<0.01 and *p<0.05; data are expressed as mean±SD.

Compared with HCG, the mean percent changes of sAA activity, pH and TPD after acid stimulation were decreased significantly in MG (*P*< 0.05, Fig 1A, 1B and 1D). Moreover, there were more cases whose percent change value less than zero in MG as compared with HCG, including sAA activity (80.00%, 48/60 vs 25.00%, 15/60, *p*< 0.0001) and pH (38.33%, 23/60 vs 13.33%, 8/60, *p* = 0.0031), but no significant difference between HCG and MG was observed in SFR, Cl^-^and Ca^2+^ concentration (Fig 1).

### Salivary biochemical characteristics in the N-group and M-group of MG patients

**Fig 2:**
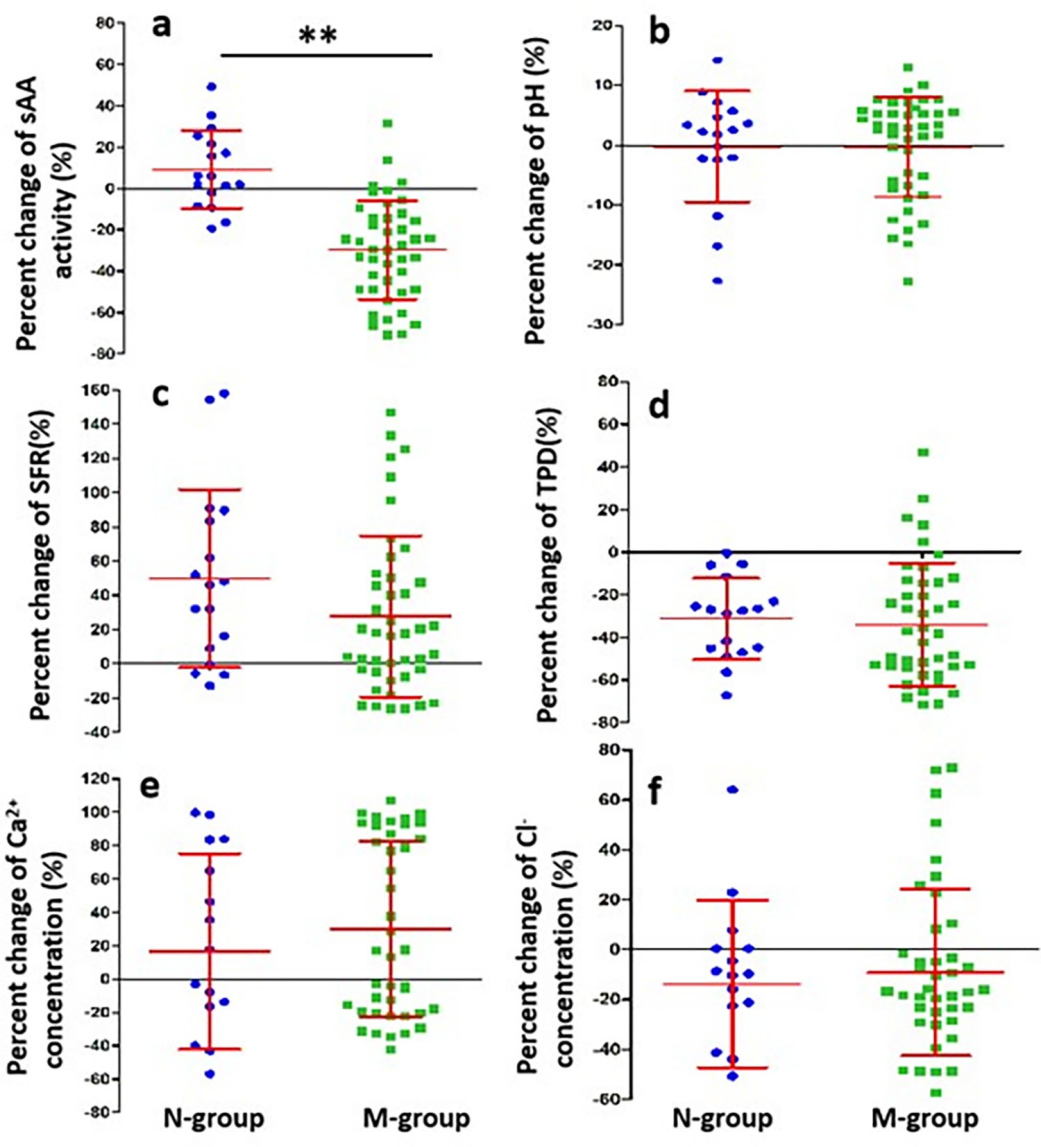
Percent changes in salivary indicators after acid stimulation in the N-group and M-group of MG. **(a)**: sAA activity, **(b)**: pH value, **(c)**: SFR, **(d)**: TPD, **(e)**: Ca^2+^ concentration, **(f)**: Cl^-^ concentration. Percent change reflects the extent of salivary secretion respond to acid stimulation and is calculated as [(value post-acid stimulation) − (value pre-acid stimulation)]/(value pre-acid stimulation) × 100%. N-group (n = 17) versus M-group (n = 43). The independent sample t test was applied at**p<0.01; data are expressed as mean ±SD.

The sAA activity was decreased significantly after acid stimulation in the M-group (*P*<0.01, Table 3), while the mean value of sAA activity was increased in the N-group although without significant difference between pre- and post-acid stimulation (*p*>0.05, Table 3). Meanwhile, the percent change in sAA activity after acid stimulation in the M-group was significantly lower than that in the N-group (*p*<0.01, Fig 2A). SFR were increased significantly compared with the value at pre-acid stimulation in the M-group and N-group (*p*<0.01, Table 3). TPD were decreased significantly compared with the value at pre-acid stimulation in the M-group (*p*<0.01, Table 3), the mean value of TPD was also decreased in the N-group although without significant difference.

**Table 3:** Salivary biochemical characteristics in the N- and M-groups of MG under both pre- and post-acid stimulation conditions.

		N	sAA activity (U/mL)	pH value	SFR (mL/min)	TPD (mg/mL)	[Cl^−^] (mg/dL)	[Ca^2+^] (mg/dL)
N-group	Pre-	17	576.80±300.32	6.38±0.56	1.02±0.53	1.83±1.11	89.22±54.91	10.39±8.49
	Post-	17	604.05±292.69	6.36±0.84	1.39±0.67*	1.19±0.70	71.47±44.16	9.69±7.61
M-group	Pre-	43	574.49±265.26	6.38±0.47	0.94±0.51	2.09±1.27	87.55±41.19	10.86±7.30
	Post-	43	390.04±204.57*§	6.37±0.77	1.10±0.54*	1.22±0.69*	72.22±29.43	12.01±7.11

Note

*p<0.01, compared with the value at pre-acid stimulation in the same group by 2×2 mixed ANOVA when variances are not significantly different or by Wilcoxon signed-rank test when variances are significantly different.

^§^*p*<0.05, compared with the value in the N-group by 2×2 mixed ANOVA when variances are not significantly different or by Mann-Whitney U test when variances are significantly different. Data are expressed as mean±SD.

### The level of sAA activity in MG patients with normal BMI or low BMI

In present cohort of MG patients, 36 were considered under-wight (BMI <18.5), 24 normal-weight (18.5 ≤ BMI < 25.0), and none was overweight (BMI > 25.0) (Table 4). No difference in sAA activity was observed between normal-weight and under-weight patients under pre- and post-acid stimulation conditions (Table 4). Compared with the number of normal-weight patients, more under-weight patients displayed the ratio of sAA activity less than 1.0 (86.11%, 31/36 vs 37.50%, 9/24, p < 0.0001), and the ratio of sAA activity decreased significantly (p < 0.01) in under-weight patients (Table 4).

**Table 1:** Comparison of sAA activity between normal- and under-weight MG patients.

	N	sAA activity (U/mL)		Ratio of sAA activity (post- VS pre-)
		Pre-acid stimulation	Post-acid stimulation	
Normal BMI	24	551.96±273.11	508.10±283.86	1.08±0.35
Low BMI	36	590.60±275.74	412.40±220.46	0.69±0.26*

Note

*p<0.01, compared with normal BMI by unpaired t-test when variances are not significantly different. The World Health Organization considers a BMI of less than 18.5 as underweight (low BMI), the BMI that ranges from 18.5 to 25.0 as healthy weight (normal BMI). The ratio of sAA activity is calculated as sAA activity value under pre-stimulation condition divided by the value under post-stimulation condition. The data were expressed as mean±SD.

### Correlation between ratio of sAA activity and BMI in HCG and MG

**Fig 3:**
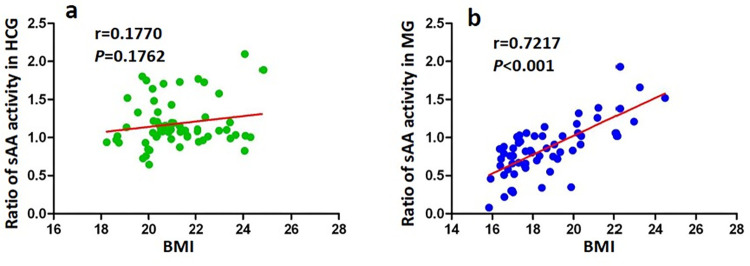
Spearman correlation analysis between the ratio of sAA activity and BMI in HCG (a) and MG patients (b).

Spearman correlation analysis showed that there was a significant strong positive correlation between the ratio of sAA activity and BMI in MG (r = 0.7217, *p*<0.001, Fig 3B), While there was no significant correlation in HCG(r = 0.1770, *p* = 0.1762, Fig 3A).

## Discussion

The present study has examined changes in saliva constituents before and after citric acid stimulation, and our data revealed significant difference in biochemical characteristics between MG and healthy controls. These differences include increase in concentrations of TPD, Ca2+and Cl−, and decrease in pH value and SFR under both pre- and post-acid stimulation conditions. Remarkably, the sAA activity responding to acid stimulation was reduced in MG patients, particularly in malnutrition subjects. These results suggested that the compositions and mechanisms of saliva secretions were abnormally altered in MG patients. We thus propose that saliva sAA activity can serve as a valuable source of information on potential biomarkers of malnutrition MG, Any changes in sAA activity certainly reflects alterations in saliva composition as a consequence of altered salivary secretion process. Thus, monitoring saliva composition may allow us to gain insight into pathophysiology of MG, and this simple and non-invasive method should be considered favorably as a clinical diagnostic tool. The present data showed clearly that, sAA activity, in response to citric acid stimulation, was markedly decreased in under-weight MG patients with high nutritional risk. These results in correlation with reduced sAA activity responding to acid stimulation, suggest that low BMI (i.e. underweight) should be considered as an important marker in malnutrition MG patients. Together, our results suggest strongly that the saliva index may help to better assess the therapeutic outcome in MG combined with typical clinical signs (e.g. weakness and fatigue of the voluntary muscles).

Saliva secretion is elevated under gustatory stimulation with acid. Consequently, rapid reaction of salivary glands to acid stimulation can provide more pathophysiological information compared to single assessment of resting saliva. In line with our previous findings [20, 21, 30], current data showed that there is no significant difference in sAA activity between HCG and MG under resting conditions, but acute sAA activity responding to acid stimulation is reduced in MG and increased in HCG. These data suggested that acute salivary responses induced by acid stimulation can reflect the physiologic state of the body in MG, such as nutritional and metabolic variations. Furthermore, salivary secretion is largely dependent upon the activation of autonomic nervous system, and acute salivary responses induced by acid stimulation is closely associated with sympathetic nerve stimulation evoked. Parasympathetic and sympathetic nerves run to the target cells in salivary glands, and the major salivary glands are supplied with parasympathetic (cholinergic) nerve fibers [31]. And sympathetic nerve stimulation evokes a protein-rich secretion, whereas parasympathetic stimulation evokes a large volume of saliva [32]. Moreover, MG is caused by a disorder of neuromuscular transmission resulting from an autoimmune lesion of cholinergic receptors. Previous studies have reported that autonomic dysfunction was involved in MG, and that the cardiac autonomic dysfunction [33, 34], sympathetic hyper-reactivity and parasympathetic insufficiency were observed in MG patients [35]. Notably, the specific alternations in salivary biochemical characteristics before acid stimulation were observed in MG in the present study. These data together are indicative of an alternated composition and flow rate of saliva that are likely attributable to autonomic dysfunction in MG. The underlying pathophysiological mechanism(s) responsible for such a dysfunction remain to be defined.

Previous studies have reported that the secretory processes in salivary glands were influenced by nutritional status [36]. For instance, fasting could result in a significant decrease in saliva secretion rate, and decrease in the concentration of phosphate and sialic acid in stimulated whole saliva [37]. Moreover, children with severe or moderate chronic protein-energy malnutrition exhibited a reduced saliva secretion rate, and lowered Ca2+ and protein secretion in stimulated saliva [38]. Thus, saliva has been considered as a useful, non-invasive matrix to assess nutritional status in humans [22]. It is well established that sAA is mainly responsible for the ‘pre-digestion’ of starch into maltose and glucose in the oral cavity, and that sAA accounts for up to 50% of salivary protein in human saliva. The maltose and glucose can act as sweet taste stimuli to interact with taste receptor type 1 (T1R) 2–T1R3 sweet taste receptor, and then evoke pleasant sweet perception and influence feeding behavior [39]. Oral glucose can significantly increase preabsorptive insulin release, known as cephalic-phase insulin release (CPIR), in rats [40] and humans [41]. CPIR plays a crucial role in glucose homeostasis [42], and thus can influence individual nutrition. In addition, food intake generally involves mastication, which preferentially stimulates output of sAA [43]. Stimulated sAA might influence individual’s appetite [44]. Moreover, the way how sAA levels are related to individual nutritional status is probably following [21]: sAA levels affect the efficiency of breaking down starch-based foods into maltose and glucose in the oral cavity; then the two sugars affect individual sweet taste perception and CPIR, which together determine starch-based food consumption (energy intake) and eventually influence individual nutritional status. Hence we hypothesized that the acute sAA activity responding to acid stimulation may be directly associated with the nutritional status. As expected, our previous studies observed a significant decrease in the ratio of sAA activity in chronic non-atrophy gastritis patients [20] and in thin children [21]. In line with the above findings, the present data have shown that sAA activity responding to acid stimulation was significantly reduced in MG, the ratio of sAA activity in MG patients with low BMI was significantly decreased compared to those with normal BMI, and a significant strong positive correlation between the ratio of sAA activity and BMI in MG. These data suggested that the acute sAA activity responding to acid stimulation may be directly associated with the nutritional status.

In addition, one issue here that we need to focus on is the effect of pH on salivary amylase, and about the effect of pH on amylase activity can be discussed in two parts, a detection phase, and a preservation phase. First, the effect of pH can be ruled out from the sAA activity detection phase in this experiment because the first step of sAA activity detection is dilution of saliva, which was diluted 2000 fold, and at such a large dilution ratio, the pH values of the diluted saliva were almost consistent (as calculated according to post dilution pH values: saliva with pH = 6 and pH = 6.9 had pH values of 6.9957, 6.9995, respectively, after 1000 fold dilution). An additional point is whether saliva of different pH causes irreversible effects on the activity of sAA during preservation. In general, pH affects enzyme activity by influencing the binding and catalysis of enzymes and substrates, the enzyme can be inactivated by strong acids and strong alkali, but the pH of saliva in this experiment is close to neutral, and it has been shown that pancreatic α- amylase is stable over a wide range of pH values (5.0–10.5) [45], moreover, the experimental concern is the result of the sAA activity of the patient, which should not be excluded, even if different pH preservation could affect the sAA activity of the patient, because this was due to the salivary microenvironment of the patient and not caused by experimental error. But how much the effect of cryopreservation at different pH values on the sAA activity of saliva is still a matter to be explored, and it is necessary to select the sAA standard solutions of the same activity, adjust to different pH values for preservation, and examine the sAA activity at different periods of preservation, but the effect of pH regulating reagents on the enzyme activity should be excluded.

Three limitations of the present study are acknowledged. First, the sample size is limited and there ware not enough patients for gender analysis. Second, this cross-sectional study lacks temporal perspective of changes in saliva composition. Future studies should compare changes in saliva composition before and after nutritional support. Third, whether or not changes in sAA activity in malnutrition MG patients are caused by malnutrition or by autonomic dysfunction, or both, in MG are speculative and merit further investigation.

## Supporting information

S1 Data Data of healthy control group. (XLSX)

S2 Data Data of MG group. (XLS)

S1 Table (DOCX)

S2 Table (DOCX)

S3 Table (DOCX)

S4 Table (DOCX)

## References

[1] Mantegazza R, Cavalcante P. Diagnosis and treatment of myasthenia gravis. Curr Opin Rheumatol 2019;31:623–633. doi: 10.1097/BOR.0000000000000647 31385879

[2] Hoesmith Nicolle. Myasthenia gravis (MG). San Diego: Plural Publishing, Inc, 2010.

[3] Serra-Prat M, Palomera M, Gomez C, Sar-Shalom D, Saiz A, Montoya JG, et al. Oropharyngeal dysphagia as a risk factor for malnutrition and lower respiratory tract infection in independently living older persons: a population-based prospective study. Age Ageing 2012;41:376–81. doi: 10.1093/ageing/afs006 22311895

[4] Ekberg O, Feinberg MJ. Altered swallowing function in elderly patients without dysphagia: radiologic findings in 56 cases. AJR. American journal of roentgenology 1991;156:1181–4. doi: 10.2214/ajr.156.6.2028863 2028863

[5] Freijer K, Bours MJL, Nuijten MJC, Poley MJ, Meijers JMM, Halfens RJG, et al. The economic value of enteral medical nutrition in the management of disease-related malnutrition: a systematic review. J Am Med Dir Assoc 2014;15:17–29. doi: 10.1016/j.jamda.2013.09.005 24239013

[6] Raynaud-Simon A, Revel-Delhom C, Hébuterne X. Clinical practice guidelines from the French Health High Authority: nutritional support strategy in protein-energy malnutrition in the elderly. Clinical nutrition (Edinburgh, Scotland) 2011;30:312–9. doi: 10.1016/j.clnu.2010.12.003 21251732

[7] Guang HS, Lei L, Qing ZH, Jin-Ping F, Xin-Hua W, Yong-Qian S. Influencing Factors of Respiratory Failure in Patients with Myasthenia Gravis and Related Factors of Prognosis. Practical Journal of Cardiac Cerebral Pneumal and Vascular Disease 2015;23:32–35.

[8] Changyi O, Hao R, Li Q, Zhidong H, Zhongqiang L, Juan D, et al. Correlation factors of 127 times pre-crisis state in patients with myasthenia gravis. National Medical Journal of China 2017;97:2884–2889. doi: 10.3760/cma.j.issn.0376-2491.2017.37.002 29050155

[9] Xin Y, Cai H, Wu L, Cui Y. The Effect of Immunonutrition on the Postoperative Complications in Thymoma with Myasthenia Gravis. Mediat Inflamm 2016:8781740. doi: 10.1155/2016/8781740 27956763 PMC5121463

[10] Lin H, Zhang H, Lin Z, Li X, Kong X, Sun G. Review of nutritional screening and assessment tools and clinical outcomes in heart failure. Heart Fail Rev 2016;21:549–65. doi: 10.1007/s10741-016-9540-0 26920682

[11] Kondrup J. Can food intake in hospitals be improved? Clin Nutr 2001;20:153–160.

[12] Kyle UG, Kossovsky MP, Karsegard VL, Pichard C. Comparison of tools for nutritional assessment and screening at hospital admission: a population study.; 2006. p. 409–17. 10.1016/j.clnu.2005.11.001 16356595

[13] Spielmann N, Wong DT. Saliva: diagnostics and therapeutic perspectives. Oral Dis 2011;17:345–54. doi: 10.1111/j.1601-0825.2010.01773.x 21122035 PMC3056919

[14] Singh PB, Young A, Homayouni A, Hove LH, Petrovski BÉ, Herlofson BB, et al. Distorted Taste and Impaired Oral Health in Patients with Sicca Complaints. Nutrients 2019;11. doi: 10.3390/nu11020264 30682880 PMC6412562

[15] Pedersen AM, Bardow A, Jensen SB, Nauntofte B. Saliva and gastrointestinal functions of taste, mastication, swallowing and digestion. Oral Dis 2002;8:117–29. doi: 10.1034/j.1601-0825.2002.02851.x 12108756

[16] Rusthen S, Young A, Herlofson BB, Aqrawi LA, Rykke M, Hove LH, et al. Oral disorders, saliva secretion, and oral health-related quality of life in patients with primary Sjögren’s syndrome. Eur J Oral Sci 2017;125:265–271. doi: 10.1111/eos.12358 28643390

[17] Ferris A, Schlitzer J, Schierberl M. Nutrition and taste and smell deficits: A risk factor or an adjustment. MA, USA: Collamore Press: Lexington, 1986.

[18] M K, J B. Interaction of the Chemical Senses with Nutrition. Academic Press 2012.

[19] A A. Oral health condition and salivary constituent (Zinc, Copper, Calcium, Iron and total Proteins) among a selected overweight primary school children: Baghdad University; 2009.

[20] Lin C, Wang L, Yang L, Qiu X, Wang D, Liang X, et al. Abnormalities in acute salivary biochemical characteristic responses to gustatory stimulation with citric acid in chronic non-atrophic gastritis. J Gastroen Hepatol 2019;34:1563–1570. doi: 10.1111/jgh.14587 30597598

[21] Chen LH, Yang ZM, Chen WW, Lin J, Zhang M, Yang XR, et al. Attenuated acute salivary alpha-amylase responses to gustatory stimulation withcitric acid in thin children. The British journal of nutrition 2015;113:1078–85. doi: 10.1017/S0007114515000446 25784372

[22] Logan D, Wallace SM, Woodside J, Mckenna G. Salivary Biomarkers of Nutritional Status: a Systematic Review. P Nutr Soc 2020;79. 10.1016/j.jdent.2021.103840 34624418

[23] Thanvi BR, Lo TCN. Update on myasthenia gravis. Postgrad Med J 2004;80:690–700. doi: 10.1136/pgmj.2004.018903 15579606 PMC1743153

[24] Jaretzki AR, Barohn RJ, Ernstoff RM, Kaminski HJ, Keesey JC, Penn AS, et al. Myasthenia gravis: recommendations for clinical research standards. Task Force of the Medical Scientific Advisory Board of the Myasthenia Gravis Foundation of America. Neurology 2000;55:16–23. doi: 10.1212/wnl.55.1.16 10891897

[25] Wang L, Lin C, Yang L, Li R, Chen L, Zhang L. Effect of 3 saliva collection methods on salivary secretion. Shanghai journal of stomatology 2015;24:563–8. 26598189

[26] Jensen SB, Vissink A. Salivary gland dysfunction and xerostomia in Sjogren’s syndrome. Oral Maxil Surg Clin 2014;26:35–53. doi: 10.1016/j.coms.2013.09.003 24287192

[27] Chicharro JL, Lucia A, Perez M, Vaquero AF, Urena R. Saliva composition and exercise. Sports medicine (Auckland, N.Z.) 1998;26:17–27. doi: 10.2165/00007256-199826010-00002 9739538

[28] Rohleder N, Nater UM. Determinants of salivary alpha-amylase in humans and methodological considerations. Psychoneuroendocrino 2009;34:469–85. doi: 10.1016/j.psyneuen.2008.12.004 19155141

[29] Kondrup J, Rasmussen HH, Hamberg O, Stanga Z. Nutritional risk screening (NRS 2002): a new method based on an analysis of controlled clinical trials. Clinical Nutrition 2003;22:321–36. doi: 10.1016/s0261-5614(02)00214-5 12765673

[30] Lihui W, Long Y, Chuanquan L, Qiuxianghong, Ruliu L, Weiwen C. Salivary analysis of chronic gastritis patients with syndrome of deficiency of spleen qi and dampness-heat due to spleen deficiency. China J. Traditional Chin. Med. Pharm 2017;32:1324–1327.

[31] Garrett JR. The proper role of nerves in salivary secretion: a review. J Dent Res 1987;66:387–97. doi: 10.1177/00220345870660020201 3305622

[32] Proctor GB, Carpenter GH. Salivary secretion: mechanism and neural regulation. Monographs in oral science 2014;24:14–29. doi: 10.1159/000358781 24862591

[33] Kocabas ZU, Kizilay F, Basarici I, Uysal H. Evaluation of cardiac autonomic functions in myasthenia gravis. Neurol Res 2018;40:405–412. doi: 10.1080/01616412.2018.1446690 29607742

[34] Peric S, Rakocevic-Stojanovic V, Nisic T, Pavlovic S, Basta I, Popovic S, et al. Cardiac autonomic control in patients with myasthenia gravis and thymoma. J Neurol Sci 2011;307:30–3. doi: 10.1016/j.jns.2011.05.028 21658726

[35] Shukla G, Gupta S, Goyal V, Singh S, Srivastava A, Behari M. Abnormal sympathetic hyper-reactivity in patients with myasthenia gravis: a prospective study. Clin Neurol Neurosur 2013;115:179–86. doi: 10.1016/j.clineuro.2012.05.013 22676958

[36] Johansson I, Ericson T, Steen L. Studies of the effect of diet on saliva secretion and caries development: the effect of fasting on saliva composition of female subjects. The Journal of nutrition 1984;114:2010–20. doi: 10.1093/jn/114.11.2010 6208345

[37] Van Lancker A, Verhaeghe S, Van Hecke A, Vanderwee K, Goossens J, Beeckman D. The association between malnutrition and oral health status in elderly in long-term care facilities: a systematic review. Int J Nurs Stud 2012;49:1568–81. doi: 10.1016/j.ijnurstu.2012.04.001 22542267

[38] Johansson I, Lenander-Lumikari M, Saellström AK. Saliva composition in Indian children with chronic protein-energy malnutrition. J Dent Res 1994;73:11–9. doi: 10.1177/00220345940730010101 8294612

[39] Bachmanov AA, Bosak NP, Floriano WB, Inoue M, Li X, Lin C, et al. Genetics of sweet taste preferences. Flavour Frag J 2011;26:286–294. doi: 10.1002/ffj.2074 21743773 PMC3130742

[40] Grill HJ, Berridge KC, Ganster DJ. Oral glucose is the prime elicitor of preabsorptive insulin secretion. The American journal of physiology 1984;246:R88–95. doi: 10.1152/ajpregu.1984.246.1.R88 6364839

[41] Mandel AL, Breslin PAS. High endogenous salivary amylase activity is associated with improved glycemic homeostasis following starch ingestion in adults. The Journal of nutrition 2012;142:853–8. doi: 10.3945/jn.111.156984 22492122 PMC3327743

[42] Ahrén B, Holst JJ. The cephalic insulin response to meal ingestion in humans is dependent on both cholinergic and noncholinergic mechanisms and is important for postprandial glycemia. Diabetes 2001;50:1030–8. doi: 10.2337/diabetes.50.5.1030 11334405

[43] Navazesh M, Kumar SKS. Measuring salivary flow: challenges and opportunities. Journal of the American Dental Association 2008;139 Suppl:35S–40S. doi: 10.14219/jada.archive.2008.0353 18460678

[44] Harthoorn LF, Dransfield E. Periprandial changes of the sympathetic-parasympathetic balance related to perceived satiety in humans. Eur J Appl Physiol 2008;102:601–8. doi: 10.1007/s00421-007-0622-5 18092177 PMC2225999

[45] Sky-Peck HH, Thuvasethakul P. Human pancreatic alpha-amylase. II. Effects of pH, substrate and ions on the activity of the enzyme. Ann Clin Lab Sci. 1977 Jul-Aug;7(4):310–7. 20029

