# Supplementary material for: Decreased salivary α-amylase activity responding to citric acid stimulation in Myasthenia gravis with malnutrition
Source: PLoS One. 2022 Jun 15;17(6):e0269621. doi: 10.1371/journal.pone.0269621 (PMC9200330; doi:10.1371/journal.pone.0269621)
Supplement: S1 Table — (DOCX) [file pone.0269621.s003.docx]

**Table 1:**

①mean±SD

| Group Statistics | | | | | |
| --- | --- | --- | --- | --- | --- |
|  | grouping1 | N | Mean | Std. Deviation | Std. Error Mean |
| Age | 1 | 60 | 30.62 | 5.764 | .744 |
|  | 2 | 60 | 42.48 | 9.247 | 1.194 |
| BMI | 1 | 60 | 21.1927 | 1.57675 | .20356 |
|  | 2 | 60 | 18.5728 | 2.04101 | .26349 |

②compare

| Tests of Normality | | | | | | | |
| --- | --- | --- | --- | --- | --- | --- | --- |
|  | grouping | Kolmogorov-Smirnova | | | Shapiro-Wilk | | |
|  |  | Statistic | df | Sig. | Statistic | df | Sig. |
| BMI | 1 | .104 | 60 | .173 | .965 | 60 | .082 |
|  | 2 | .152 | 60 | .001 | .902 | 60 | .000 |
| age | 1 | .275 | 60 | .000 | .857 | 60 | .000 |
|  | 2 | .210 | 60 | .000 | .806 | 60 | .000 |
| a. Lilliefors Significance Correction | | | | | | | |

Nonparametric test

NOTE：
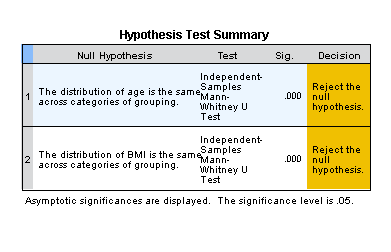


The results of nonparametric test showed that there were significant differences in age and BMI between HCG and MG groups.
