# Supplementary material for: Decreased salivary α-amylase activity responding to citric acid stimulation in Myasthenia gravis with malnutrition
Source: PLoS One. 2022 Jun 15;17(6):e0269621. doi: 10.1371/journal.pone.0269621 (PMC9200330; doi:10.1371/journal.pone.0269621)
Supplement: S2 Table — (DOCX) [file pone.0269621.s004.docx]

**Table 2:**

①mean±SD

| Group Statistics | | | | | |
| --- | --- | --- | --- | --- | --- |
|  | grouping1 | N | Mean | Std. Deviation | Std. Error Mean |
| sAAM | 1 | 60 | 575.1423 | 273.03317 | 35.24843 |
|  | 2 | 60 | 450.6791 | 249.95532 | 32.26909 |
| sAAH | 1 | 60 | 505.3857 | 308.32622 | 39.80474 |
|  | 2 | 60 | 542.0387 | 284.38599 | 36.71407 |
| PHM | 1 | 60 | 6.3795 | .49078 | .06336 |
|  | 2 | 60 | 6.3683 | .78433 | .10126 |
| PHH | 1 | 60 | 6.7510 | .32893 | .04246 |
|  | 2 | 60 | 6.9268 | .26666 | .03443 |
| SFRM | 1 | 60 | .9598 | .51259 | .06618 |
|  | 2 | 60 | 1.1777 | .59018 | .07619 |
| SFRH | 1 | 60 | 1.3033 | .47992 | .06196 |
|  | 2 | 60 | 1.6523 | .57645 | .07442 |
| TPDM | 1 | 60 | 2.0166 | 1.22364 | .15797 |
|  | 2 | 60 | 1.2104 | .68459 | .08838 |
| TPDH | 1 | 60 | 1.2317 | .78522 | .10137 |
|  | 2 | 60 | .8990 | .62161 | .08025 |
| Ca^2+^M | 1 | 57 | 10.7284 | 7.57534 | 1.00338 |
|  | 2 | 57 | 11.3626 | 7.26242 | .96193 |
| Ca^2+^H | 1 | 60 | 4.2427 | 3.05053 | .39382 |
|  | 2 | 60 | 4.4603 | 2.29253 | .29596 |
| Cl^-^M | 1 | 57 | 88.0149 | 44.94687 | 5.95336 |
|  | 2 | 57 | 72.0093 | 33.77884 | 4.47412 |
| Cl^-^H | 1 | 60 | 51.4557 | 26.44874 | 3.41452 |
|  | 2 | 60 | 48.2503 | 23.74474 | 3.06543 |

②Homogeneity of variance test between groups

| Test of Homogeneity of Variance | | | | | |
| --- | --- | --- | --- | --- | --- |
|  | | Levene Statistic | df1 | df2 | Sig. |
| sAAactivity | Based on Mean | 1.397 | 3 | 230 | .244 |
|  | Based on Median | 1.084 | 3 | 230 | .357 |
|  | Based on Median and with adjusted df | 1.084 | 3 | 227.974 | .357 |
|  | Based on trimmed mean | 1.465 | 3 | 230 | .225 |
| PH | Based on Mean | 26.214 | 3 | 230 | .000 |
|  | Based on Median | 18.902 | 3 | 230 | .000 |
|  | Based on Median and with adjusted df | 18.902 | 3 | 138.256 | .000 |
|  | Based on trimmed mean | 24.912 | 3 | 230 | .000 |
| SFR | Based on Mean | 1.040 | 3 | 230 | .376 |
|  | Based on Median | 1.050 | 3 | 230 | .371 |
|  | Based on Median and with adjusted df | 1.050 | 3 | 224.297 | .371 |
|  | Based on trimmed mean | 1.071 | 3 | 230 | .362 |
| TPD | Based on Mean | 10.014 | 3 | 230 | .000 |
|  | Based on Median | 9.108 | 3 | 230 | .000 |
|  | Based on Median and with adjusted df | 9.108 | 3 | 182.681 | .000 |
|  | Based on trimmed mean | 9.467 | 3 | 230 | .000 |
| Cl | Based on Mean | 8.320 | 3 | 230 | .000 |
|  | Based on Median | 6.842 | 3 | 230 | .000 |
|  | Based on Median and with adjusted df | 6.842 | 3 | 189.058 | .000 |
|  | Based on trimmed mean | 7.912 | 3 | 230 | .000 |
| Ca^2+^ | Based on Mean | 37.428 | 3 | 230 | .000 |
|  | Based on Median | 33.094 | 3 | 230 | .000 |
|  | Based on Median and with adjusted df | 33.094 | 3 | 159.097 | .000 |
|  | Based on trimmed mean | 36.187 | 3 | 230 | .000 |

NOTE：As can be seen from the above results, only sAA activity and SFR were homogeneous between groups and suitable for 2×2 mixed ANOVA, others used nonparametric tests, and the results were consistent with the previous ones.

③2×2 mixed ANOVA

**sAA**


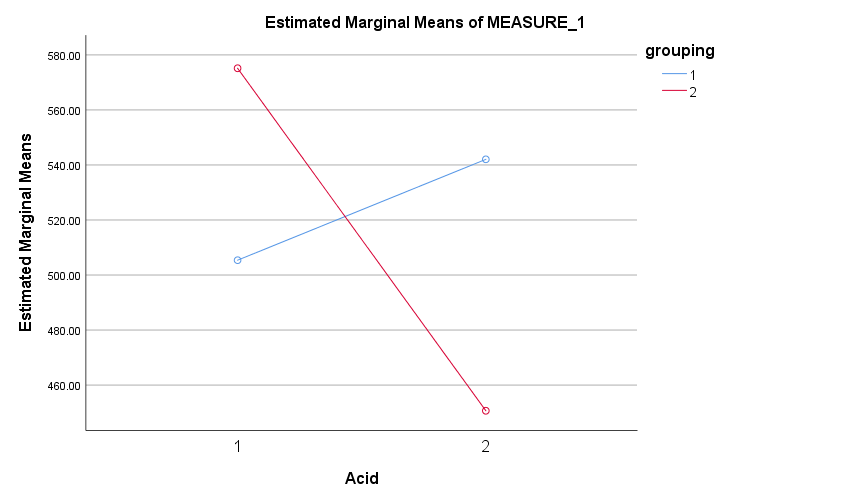


| Tests of Within-Subjects Effects | | | | | | |
| --- | --- | --- | --- | --- | --- | --- |
| Measure: MEASURE_1 | | | | | | |
| Source | | Type III Sum of Squares | df | Mean Square | F | Sig. |
| Acid | Sphericity Assumed | 115662.454 | 1 | 115662.454 | 6.708 | .011 |
|  | Greenhouse-Geisser | 115662.454 | 1.000 | 115662.454 | 6.708 | .011 |
|  | Huynh-Feldt | 115662.454 | 1.000 | 115662.454 | 6.708 | .011 |
|  | Lower-bound | 115662.454 | 1.000 | 115662.454 | 6.708 | .011 |
| Acid * grouping | Sphericity Assumed | 389381.927 | 1 | 389381.927 | 22.583 | .000 |
|  | Greenhouse-Geisser | 389381.927 | 1.000 | 389381.927 | 22.583 | .000 |
|  | Huynh-Feldt | 389381.927 | 1.000 | 389381.927 | 22.583 | .000 |
|  | Lower-bound | 389381.927 | 1.000 | 389381.927 | 22.583 | .000 |
| Error(Acid) | Sphericity Assumed | 2034618.062 | 118 | 17242.526 |  |  |
|  | Greenhouse-Geisser | 2034618.062 | 118.000 | 17242.526 |  |  |
|  | Huynh-Feldt | 2034618.062 | 118.000 | 17242.526 |  |  |
|  | Lower-bound | 2034618.062 | 118.000 | 17242.526 |  |  |

Interactions were significant and simple effects analysis was performed.→


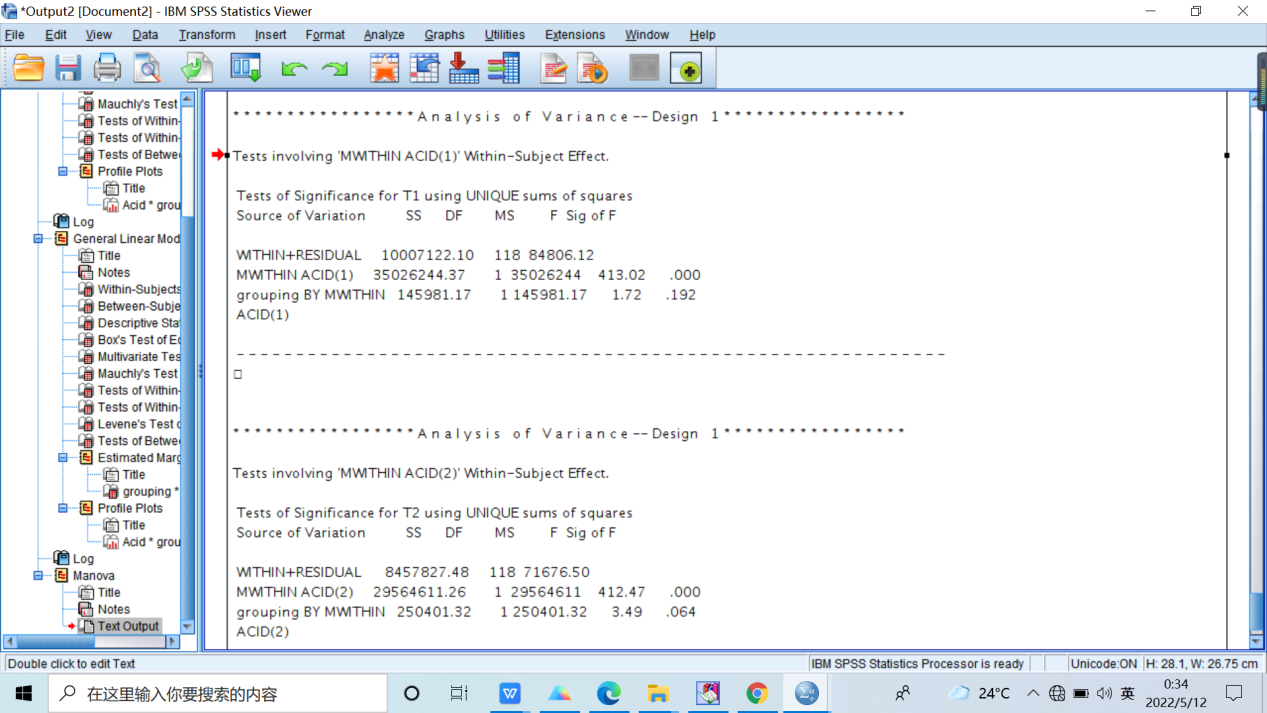


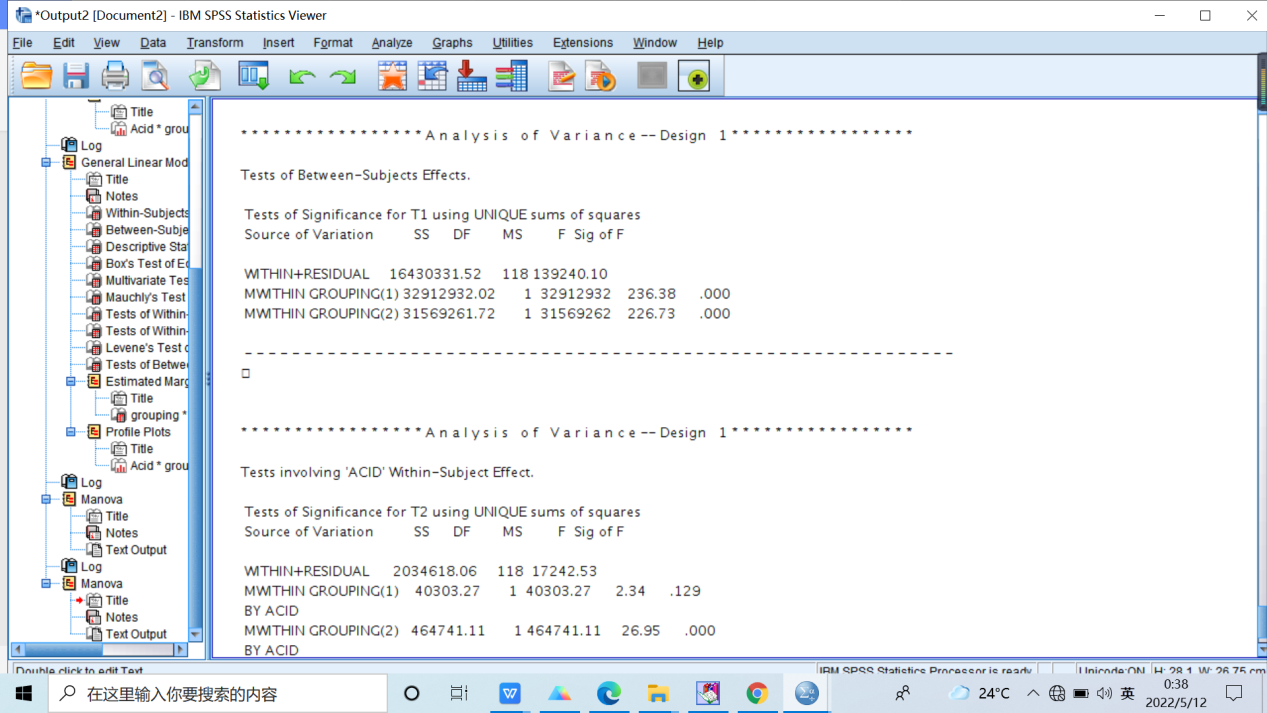


The results showed that only MG group showed significant differences in sAA activity between pre-acid and post-acid (P < 0.01), which was consistent with the previous analysis.

**SFR：**


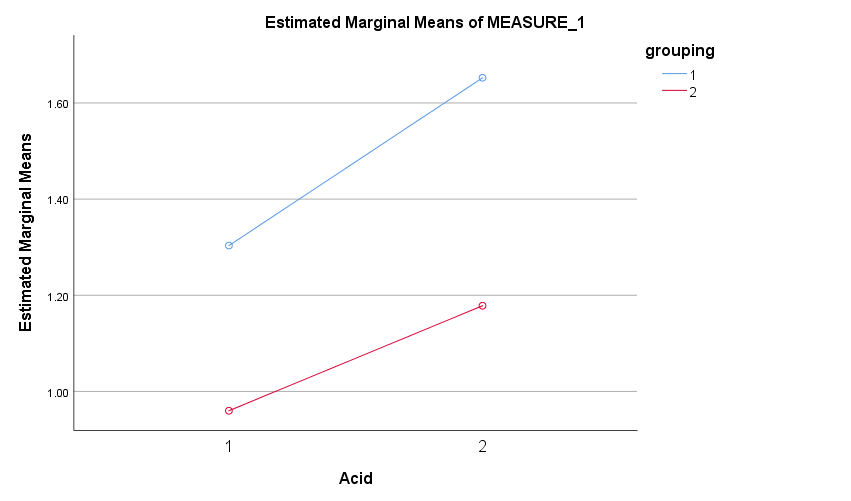


| Tests of Within-Subjects Effects | | | | | | | |
| --- | --- | --- | --- | --- | --- | --- | --- |
| Measure: MEASURE_1 | | | | | | | |
| Source | | Type III Sum of Squares | df | Mean Square | F | Sig. | Partial Eta Squared |
| Acid | Sphericity Assumed | 4.828 | 1 | 4.828 | 77.642 | .000 | .397 |
|  | Greenhouse-Geisser | 4.828 | 1.000 | 4.828 | 77.642 | .000 | .397 |
|  | Huynh-Feldt | 4.828 | 1.000 | 4.828 | 77.642 | .000 | .397 |
|  | Lower-bound | 4.828 | 1.000 | 4.828 | 77.642 | .000 | .397 |
| Acid * grouping | Sphericity Assumed | .256 | 1 | .256 | 4.119 | .045 | .034 |
|  | Greenhouse-Geisser | .256 | 1.000 | .256 | 4.119 | .045 | .034 |
|  | Huynh-Feldt | .256 | 1.000 | .256 | 4.119 | .045 | .034 |
|  | Lower-bound | .256 | 1.000 | .256 | 4.119 | .045 | .034 |
| Error(Acid) | Sphericity Assumed | 7.338 | 118 | .062 |  |  |  |
|  | Greenhouse-Geisser | 7.338 | 118.000 | .062 |  |  |  |
|  | Huynh-Feldt | 7.338 | 118.000 | .062 |  |  |  |
|  | Lower-bound | 7.338 | 118.000 | .062 |  |  |  |

Interactions were significant and simple effects analysis was performed.→


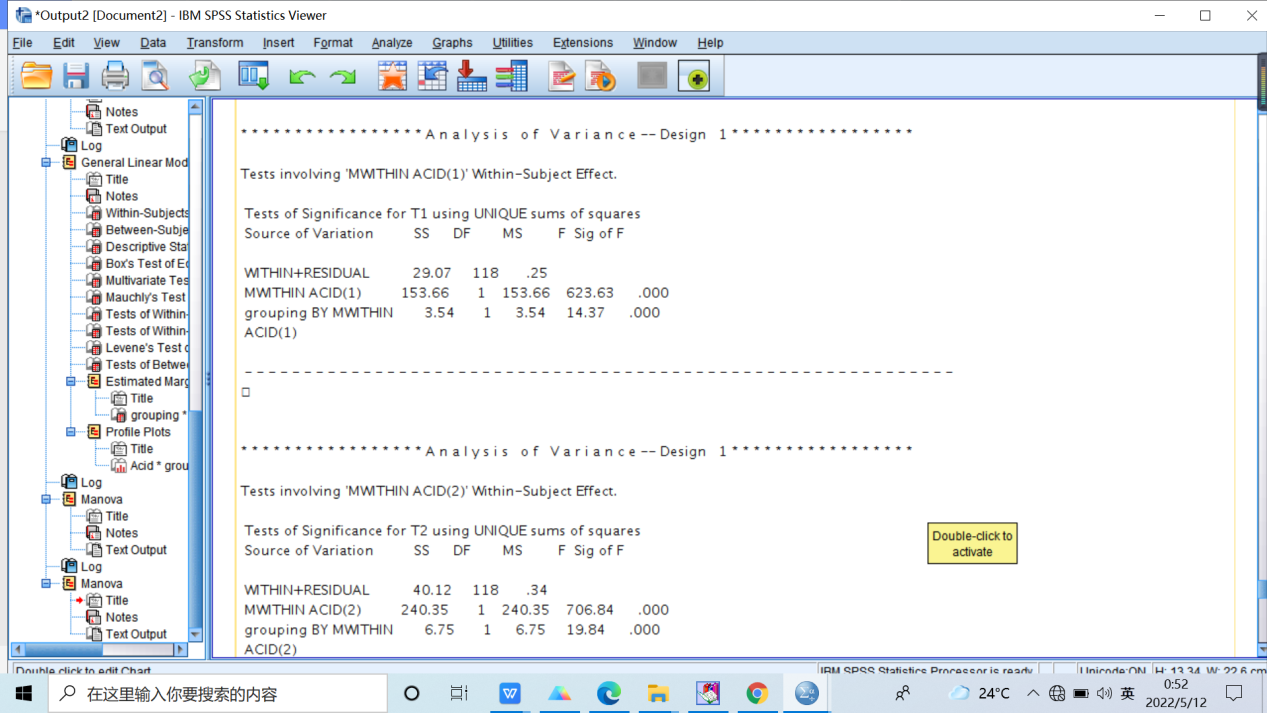


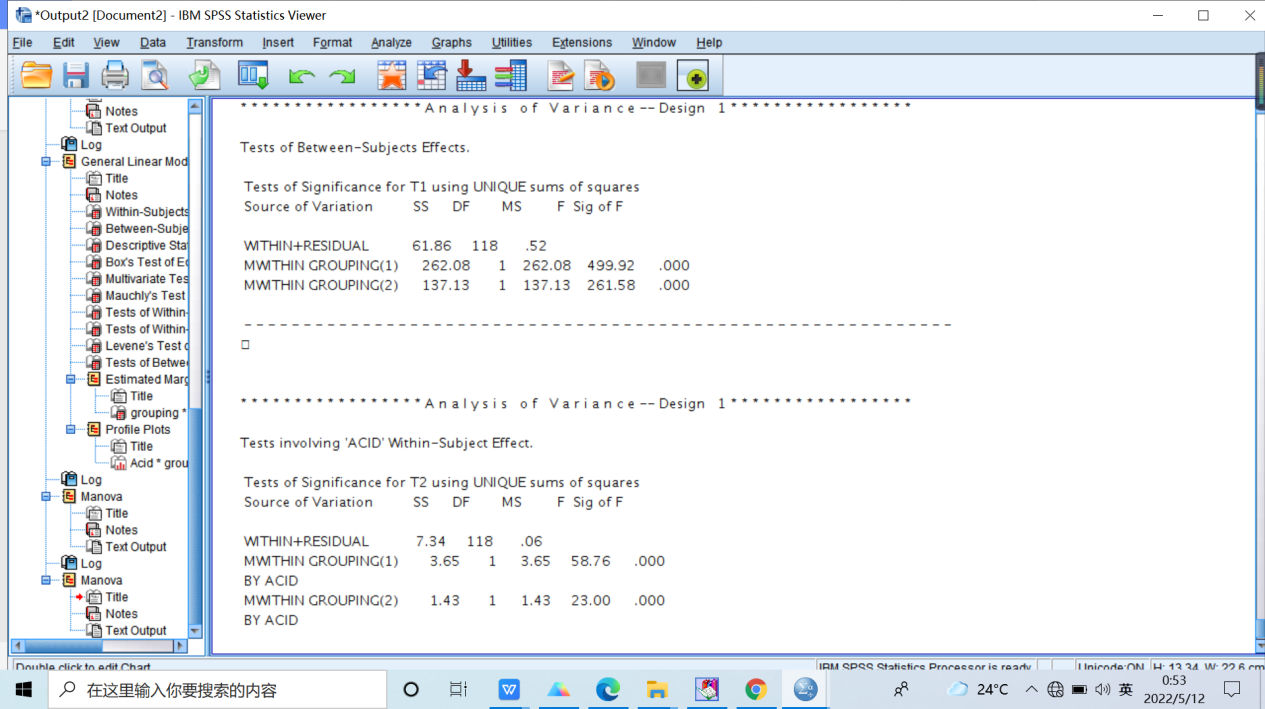


The results showed significant differences within each group for SFR and were consistent with the pre post statistics.
