# Supplementary material for: Decreased salivary α-amylase activity responding to citric acid stimulation in Myasthenia gravis with malnutrition
Source: PLoS One. 2022 Jun 15;17(6):e0269621. doi: 10.1371/journal.pone.0269621 (PMC9200330; doi:10.1371/journal.pone.0269621)
Supplement: S3 Table — (DOCX) [file pone.0269621.s005.docx]

**Table 3:**

①mean±SD

| Group Statistics | | | | | |
| --- | --- | --- | --- | --- | --- |
|  | grouping2 | N | Mean | Std. Deviation | Std. Error Mean |
| MGsAA1 | 1 | 17 | 576.7959 | 300.31716 | 72.83761 |
|  | 2 | 43 | 574.4891 | 265.25696 | 40.45131 |
| MGsAA2 | 1 | 17 | 604.0459 | 292.68602 | 70.98679 |
|  | 2 | 43 | 390.0447 | 204.56594 | 31.19601 |
| MGPH1 | 1 | 17 | 6.3776 | .55975 | .13576 |
|  | 2 | 43 | 6.3802 | .46797 | .07136 |
| MGPH2 | 1 | 17 | 6.3647 | .83912 | .20352 |
|  | 2 | 43 | 6.3698 | .77196 | .11772 |
| MGSFR1 | 1 | 17 | 1.0182 | .52890 | .12828 |
|  | 2 | 43 | .9367 | .51010 | .07779 |
| MGSFR2 | 1 | 17 | 1.3853 | .66637 | .16162 |
|  | 2 | 43 | 1.0963 | .54328 | .08285 |
| MGTPD1 | 1 | 17 | 1.8324 | 1.11876 | .27134 |
|  | 2 | 43 | 2.0900 | 1.26690 | .19320 |
| MGTPD2 | 1 | 17 | 1.1853 | .69705 | .16906 |
|  | 2 | 43 | 1.2200 | .68774 | .10488 |
| MGCa^2+^1 | 1 | 16 | 10.3906 | 8.49263 | 2.12316 |
|  | 2 | 41 | 10.8607 | 7.29571 | 1.13940 |
| MGCa^2+^2 | 1 | 16 | 9.6931 | 7.60631 | 1.90158 |
|  | 2 | 41 | 12.0137 | 7.11348 | 1.11094 |
| MGCl^-^1 | 1 | 16 | 89.2206 | 54.90907 | 13.72727 |
|  | 2 | 41 | 87.5456 | 41.19267 | 6.43321 |
| MGCl^-^2 | 1 | 16 | 71.4744 | 44.16181 | 11.04045 |
|  | 2 | 41 | 72.2180 | 29.42671 | 4.59568 |

②Homogeneity of variance test between groups

| Test of Homogeneity of Variance | | | | | |
| --- | --- | --- | --- | --- | --- |
|  | | Levene Statistic | df1 | df2 | Sig. |
| sAAactivity | Based on Mean | 2.280 | 3 | 110 | .083 |
|  | Based on Median | 1.778 | 3 | 110 | .156 |
|  | Based on Median and with adjusted df | 1.778 | 3 | 103.350 | .156 |
|  | Based on trimmed mean | 2.321 | 3 | 110 | .079 |
| PH | Based on Mean | 4.270 | 3 | 110 | .007 |
|  | Based on Median | 2.774 | 3 | 110 | .045 |
|  | Based on Median and with adjusted df | 2.774 | 3 | 91.454 | .046 |
|  | Based on trimmed mean | 3.978 | 3 | 110 | .010 |
| SFR | Based on Mean | .251 | 3 | 110 | .860 |
|  | Based on Median | .186 | 3 | 110 | .906 |
|  | Based on Median and with adjusted df | .186 | 3 | 97.222 | .906 |
|  | Based on trimmed mean | .227 | 3 | 110 | .878 |
| TPD | Based on Mean | 4.061 | 3 | 110 | .009 |
|  | Based on Median | 3.150 | 3 | 110 | .028 |
|  | Based on Median and with adjusted df | 3.150 | 3 | 79.449 | .030 |
|  | Based on trimmed mean | 3.849 | 3 | 110 | .012 |
| Ca^2+^ | Based on Mean | .517 | 3 | 110 | .671 |
|  | Based on Median | .301 | 3 | 110 | .824 |
|  | Based on Median and with adjusted df | .301 | 3 | 107.343 | .824 |
|  | Based on trimmed mean | .460 | 3 | 110 | .711 |
| Cl^-^ | Based on Mean | 3.741 | 3 | 110 | .013 |
|  | Based on Median | 3.324 | 3 | 110 | .022 |
|  | Based on Median and with adjusted df | 3.324 | 3 | 97.981 | .023 |
|  | Based on trimmed mean | 3.683 | 3 | 110 | .014 |

NOTE：As can be seen from the above results, only sAA activity、SFR and Ca^+^ were homogeneous between groups and suitable for 2×2 mixed ANOVA, others used nonparametric tests, and the results were consistent with the previous ones.

③2×2 mixed ANOVA

**sAA**


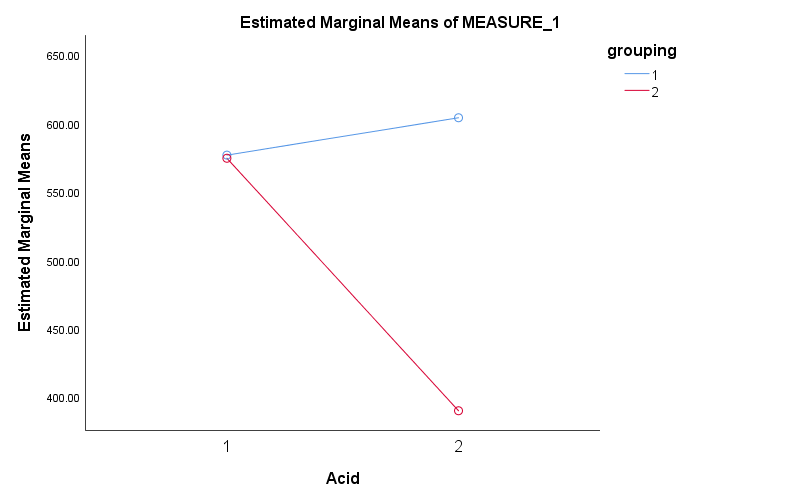


| Tests of Within-Subjects Effects | | | | | | | |
| --- | --- | --- | --- | --- | --- | --- | --- |
| Measure: MEASURE_1 | | | | | | | |
| Source | | Type III Sum of Squares | df | Mean Square | F | Sig. | Partial Eta Squared |
| Acid | Sphericity Assumed | 150525.603 | 1 | 150525.603 | 6.230 | .015 | .097 |
|  | Greenhouse-Geisser | 150525.603 | 1.000 | 150525.603 | 6.230 | .015 | .097 |
|  | Huynh-Feldt | 150525.603 | 1.000 | 150525.603 | 6.230 | .015 | .097 |
|  | Lower-bound | 150525.603 | 1.000 | 150525.603 | 6.230 | .015 | .097 |
| Acid * grouping | Sphericity Assumed | 272995.160 | 1 | 272995.160 | 11.299 | .001 | .163 |
|  | Greenhouse-Geisser | 272995.160 | 1.000 | 272995.160 | 11.299 | .001 | .163 |
|  | Huynh-Feldt | 272995.160 | 1.000 | 272995.160 | 11.299 | .001 | .163 |
|  | Lower-bound | 272995.160 | 1.000 | 272995.160 | 11.299 | .001 | .163 |
| Error(Acid) | Sphericity Assumed | 1401381.063 | 58 | 24161.742 |  |  |  |
|  | Greenhouse-Geisser | 1401381.063 | 58.000 | 24161.742 |  |  |  |
|  | Huynh-Feldt | 1401381.063 | 58.000 | 24161.742 |  |  |  |
|  | Lower-bound | 1401381.063 | 58.000 | 24161.742 |  |  |  |

Interactions were significant and simple effects analysis was performed.→


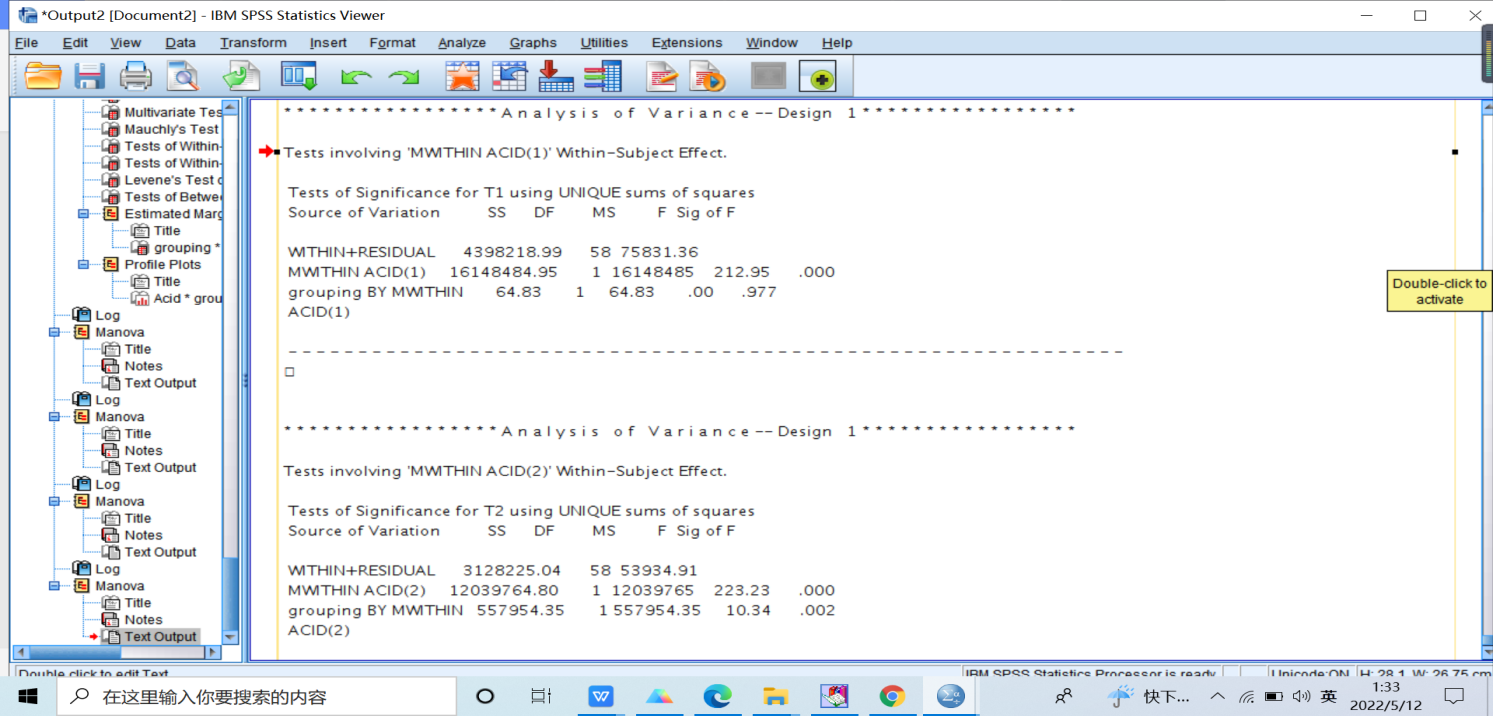


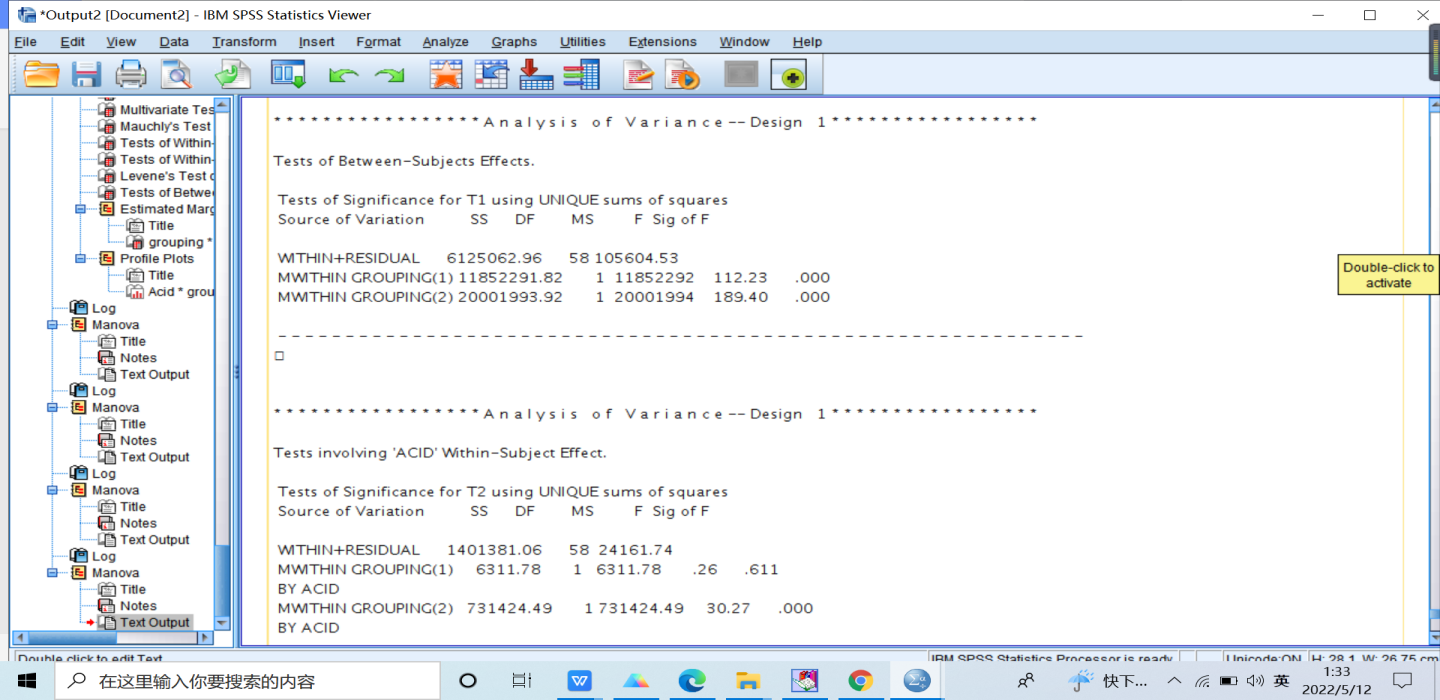


The results showed that there was a significant difference between N-group and M-group after acid (ACID2) (P = 0.002), and there was a significant difference between M-group (GROUPING2) before and after acid (P = 0.000). It is consistent with the previous analysis results.

**SFR：**

**
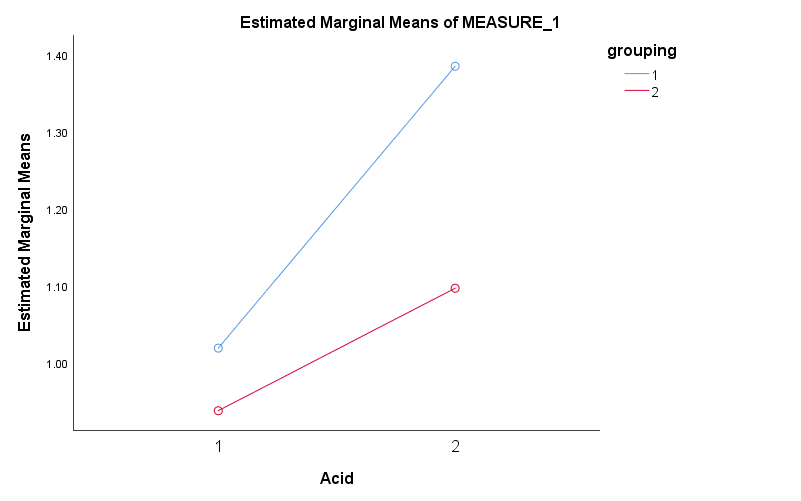
**

| Tests of Within-Subjects Effects | | | | | | | |
| --- | --- | --- | --- | --- | --- | --- | --- |
| Measure: MEASURE_1 | | | | | | | |
| Source | | Type III Sum of Squares | df | Mean Square | F | Sig. | Partial Eta Squared |
| Acid | Sphericity Assumed | 1.689 | 1 | 1.689 | 29.714 | .000 | .339 |
|  | Greenhouse-Geisser | 1.689 | 1.000 | 1.689 | 29.714 | .000 | .339 |
|  | Huynh-Feldt | 1.689 | 1.000 | 1.689 | 29.714 | .000 | .339 |
|  | Lower-bound | 1.689 | 1.000 | 1.689 | 29.714 | .000 | .339 |
| Acid * grouping | Sphericity Assumed | .262 | 1 | .262 | 4.615 | .036 | .074 |
|  | Greenhouse-Geisser | .262 | 1.000 | .262 | 4.615 | .036 | .074 |
|  | Huynh-Feldt | .262 | 1.000 | .262 | 4.615 | .036 | .074 |
|  | Lower-bound | .262 | 1.000 | .262 | 4.615 | .036 | .074 |
| Error(Acid) | Sphericity Assumed | 3.297 | 58 | .057 |  |  |  |
|  | Greenhouse-Geisser | 3.297 | 58.000 | .057 |  |  |  |
|  | Huynh-Feldt | 3.297 | 58.000 | .057 |  |  |  |
|  | Lower-bound | 3.297 | 58.000 | .057 |  |  |  |

Interactions were significant and simple effects analysis was performed.→


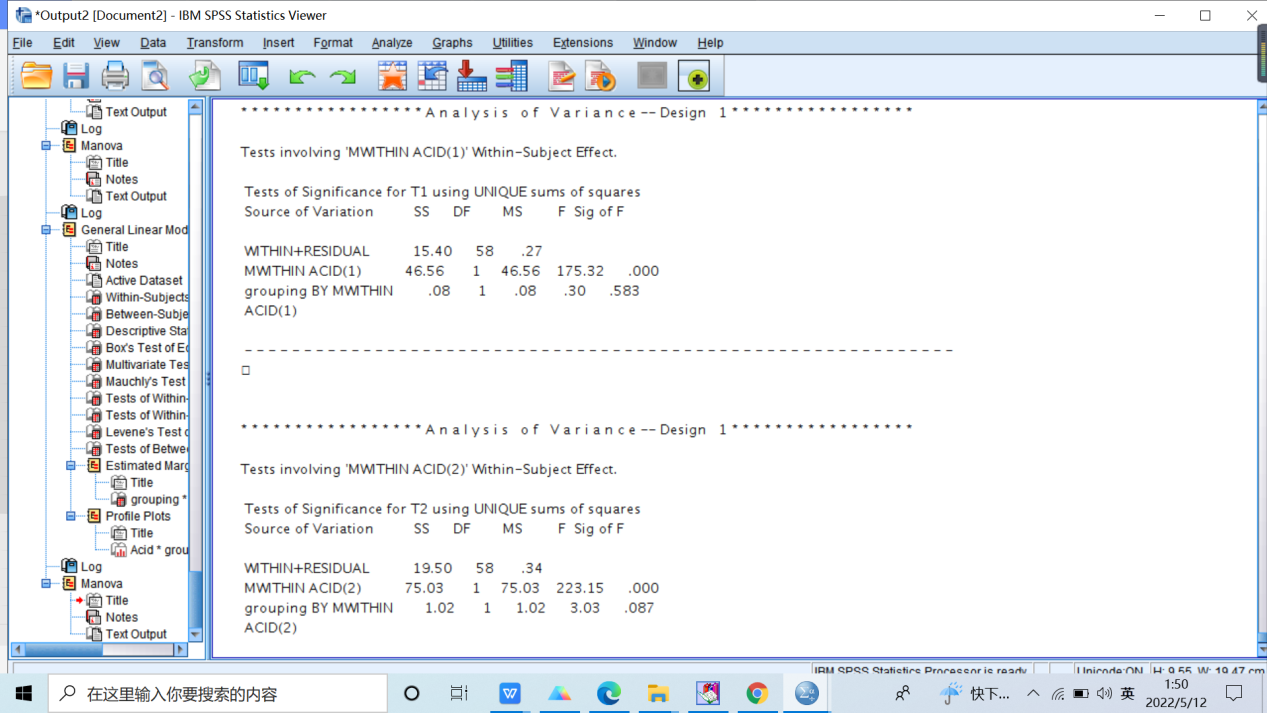


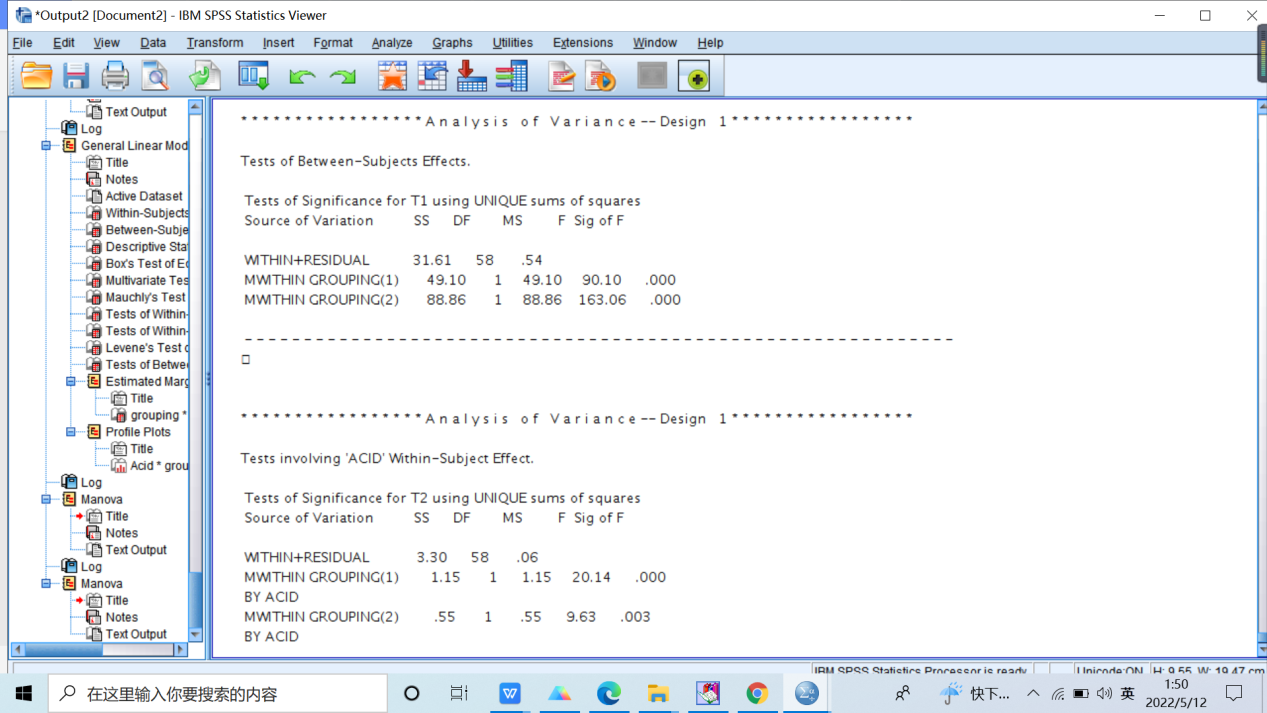


The results showed that there were significant differences in N-group (grouping2) and M-group (grouping2) before and after acid stimulation (P = 0.000, P = 0.003).This result and the previous analysis were not consistent, but the trend was consistent and did not affect the analysis and conclusions, speculating that this phenomenon might be caused by the choice of different statistical methods.

**Ca^2+^：**


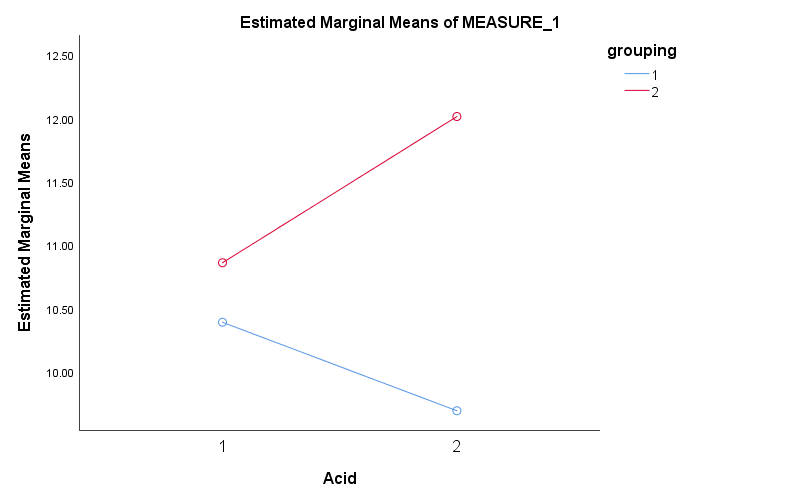


| Tests of Within-Subjects Effects | | | | | | | |
| --- | --- | --- | --- | --- | --- | --- | --- |
| Measure: MEASURE_1 | | | | | | | |
| Source | | Type III Sum of Squares | df | Mean Square | F | Sig. | Partial Eta Squared |
| Acid | Sphericity Assumed | 1.194 | 1 | 1.194 | .073 | .788 | .001 |
|  | Greenhouse-Geisser | 1.194 | 1.000 | 1.194 | .073 | .788 | .001 |
|  | Huynh-Feldt | 1.194 | 1.000 | 1.194 | .073 | .788 | .001 |
|  | Lower-bound | 1.194 | 1.000 | 1.194 | .073 | .788 | .001 |
| Acid * grouping | Sphericity Assumed | 19.703 | 1 | 19.703 | 1.200 | .278 | .021 |
|  | Greenhouse-Geisser | 19.703 | 1.000 | 19.703 | 1.200 | .278 | .021 |
|  | Huynh-Feldt | 19.703 | 1.000 | 19.703 | 1.200 | .278 | .021 |
|  | Lower-bound | 19.703 | 1.000 | 19.703 | 1.200 | .278 | .021 |
| Error(Acid) | Sphericity Assumed | 902.770 | 55 | 16.414 |  |  |  |
|  | Greenhouse-Geisser | 902.770 | 55.000 | 16.414 |  |  |  |
|  | Huynh-Feldt | 902.770 | 55.000 | 16.414 |  |  |  |
|  | Lower-bound | 902.770 | 55.000 | 16.414 |  |  |  |

The interaction was not significant (P = 0.278) and there was no difference within groups.
