# Supplementary material for: Decreased salivary α-amylase activity responding to citric acid stimulation in Myasthenia gravis with malnutrition
Source: PLoS One. 2022 Jun 15;17(6):e0269621. doi: 10.1371/journal.pone.0269621 (PMC9200330; doi:10.1371/journal.pone.0269621)
Supplement: S4 Table — (DOCX) [file pone.0269621.s006.docx]

**Table 4:**

①mean±SD

| Group Statistics | | | | | |
| --- | --- | --- | --- | --- | --- |
|  | grouping3 | N | Mean | Std. Deviation | Std. Error Mean |
| MGsAAQ | 1.00 | 24 | 551.9579 | 273.11248 | 55.74885 |
|  | 2.00 | 36 | 590.5992 | 275.74329 | 45.95722 |
| MGsAAH | 1.00 | 24 | 508.1029 | 283.85507 | 57.94167 |
|  | 2.00 | 36 | 412.3953 | 220.45932 | 36.74322 |
| MGsAAratio | 1.00 | 24 | 1.0775 | .35290 | .07204 |
|  | 2.00 | 36 | .6939 | .25545 | .04257 |

②Independent Samples Test

| Independent Samples Test | | | | | | | | | | |
| --- | --- | --- | --- | --- | --- | --- | --- | --- | --- | --- |
|  | | Levene's Test for Equality of Variances | | t-test for Equality of Means | | | | | | |
|  |  | F | Sig. | t | df | Sig. (2-tailed) | Mean Difference | Std. Error Difference | 95% Confidence Interval of the Difference | |
|  |  |  |  |  |  |  |  |  | Lower | Upper |
| MGsAAQ | Equal variances assumed | .013 | .908 | -.534 | 58 | .596 | -38.64125 | 72.39061 | -183.54680 | 106.26430 |
|  | Equal variances not assumed |  |  | -.535 | 49.776 | .595 | -38.64125 | 72.24957 | -183.77495 | 106.49245 |
| MGsAAH | Equal variances assumed | 2.220 | .142 | 1.467 | 58 | .148 | 95.70764 | 65.23495 | -34.87430 | 226.28958 |
|  | Equal variances not assumed |  |  | 1.395 | 40.874 | .171 | 95.70764 | 68.60978 | -42.86559 | 234.28087 |
| MGsAAratio | Equal variances assumed | 1.793 | .186 | 4.886 | 58 | .000 | .38361 | .07851 | .22645 | .54077 |
|  | Equal variances not assumed |  |  | 4.584 | 38.766 | .000 | .38361 | .08368 | .21433 | .55290 |

The results showed that there was significant difference in ratio of sAA activity between normal BMI and low BMI (P = 0.000).It is consistent with the previous analysis results.
